# Lemierre's syndrome and genetic polymorphisms: a case report

**DOI:** 10.1186/1471-2334-6-115

**Published:** 2006-07-17

**Authors:** Jean-Michel Constantin, Jean-Paul Mira, Renaud Guerin, Sophie Cayot-Constantin, Olivier Lesens, Florence Gourdon, Jean-Pierre Romaszko, Philippe Linval, Henri Laurichesse, Jean-Etienne Bazin

**Affiliations:** 1 Adult Intensive Care Unit, Department of anesthesiology and intensive care, University Hospital of Clermont-Ferrand, Hotel-Dieu Hospital, F-63058 Clermont-Ferrand, France; 2 Medical Intensive Care Unit and Cochin Institute INSERM U567, Cochin Saint-Vincent de Paul University Hospital, Paris, France; 3 Infectious Diseases and Tropical Medicine Department, University Hospital of Clermont-Ferrand, Hotel-Dieu Hospital, F-63058 Clermont-Ferrand, France; 4 Laboratory of Bacteriology, University Hospital of Clermont-Ferrand, F-63000 Clermont-Ferrand, France; 5 Intensive Care Unit, Moulins-Yzeure Hospital, Moulins, France

## Abstract

**Background:**

Lemierre's syndrome presents a classic clinical picture, the pathophysiology of which remains obscure. Attempts have been made to trace genetic predispositions that modify the host detection of pathogen or the resultant systemic reaction.

**Case presentation:**

A 17-year old female, with no previous medical history, was admitted to the intensive care unit for septic shock, acute respiratory distress syndrome and Lemierre's syndrome. Her DNA was assayed for single nucleotide polymorphisms previously incriminated in the detection of the pathogen, the inflammatory response and the coagulation cascade. We observed functional variations in her Toll like 5 receptor (TLR 5) gene and two coagulation variations (Tissue Factor (TF) 603 and Plasminogen-Activator-Inhibitor-1 (PAI-1) 4G-4G homozygosity) associated with thrombotic events.

**Conclusion:**

The innate immune response and the prothrombogenic mutations could explain, at least in part, the symptoms of Lemierre's syndrome. Genomic study of several patients with Lemierre's syndrome may reveal its pathophysiology.

## Background

Lemierre's syndrome presents a classic [1] but exceptional clinical picture (0.8 per million people per year) [2], the physiopathology of which remains obscure. Over the last few years, there has been a noticeable increase in efforts to identify genetic predispositions that modify the detection of the pathogen by the host or the resultant systemic reaction, efforts that may enhance our understanding of this syndrome. This article describes a young woman with Lemierre's syndrome who was found to carry several genetic polymorphisms: a single nucleotide polymorphism (SNP) in the TLR 5, and SNPs on the gene promoters coding for TF and PAI-1.

## Case presentation

A 17-year old female with no previous medical history was admitted to the emergency room of a general hospital due to a deterioration of her general condition, with fever, dyspnea and a paroxysmal severe cough. Two weeks previously, she had presented with an acute pharyngitis, which was treated with amoxicillin for 8 days. At admission, she presented with persistent lateral cervical pain, hyperleukocytosis (leukocyte count of 13,300/m^-3^, including 90% neutrophils) and a CRP at 450 mg/L, combined with an X-ray shadow on the bottom left lung. This led to a diagnosis of community acquired pneumonia. Blood samples were collected for culture and the patient benefited from treatment with amoxicillin/clavulanic acid and erythromycin.

**Figure 1:**
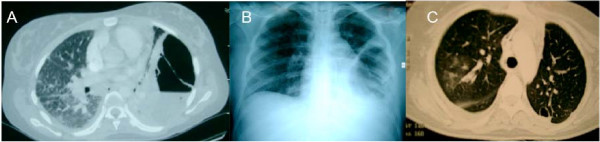
Initial thoracic CT-scan (A) and chest X-ray (B), thoracic CT-scan after chest tube and under mechanical ventilation (C).

After 48 hours in hospital, the blood cultures were positive for anaerobic Gram negative bacilli. Her antibiotic therapy was modified, to a combination of cefotaxime, gentamicin and metronizadole. Following the appearance of pain in the two hypochondria, a thoraco-abdominal CT scan was performed, which revealed a voluminous build-up of air and fluid in the left thorax (Fig 1A) and a heterogeneous hepato-splenomegaly. Within a few hours, the patient presented with a body temperature of 39°7, respiratory failure with hypotension and awareness disorders, requiring the use of mechanical ventilation and vascular filling with crystalloids followed by the administration of norepinephrine (1 μg/kg/min). She was diagnosed with Lemierre's syndrome and transferred to the intensive care unit of a university hospital center.

At admission to the intensive care unit, the patient presented with severe ARDS, with a PaO_2_/FiO_2 _ratio at 65 mmHg, PEEP14, bilateral alveolo-interstitial syndrome, left pyopneumothorax on chest x-ray (Fig 1B) and septic shock. Biologically, her CRP was 475 mg/L and her procalcitonine was 92 mg/L. A left thoracic drain was installed, which removed 350 ml of the purulent liquid. Mechanical ventilation was implemented according to the recommendations of the National Institutes of Health. Her antibiotic regimen was modified to a combination of imipenem/cilastatin (3000 mg/day) and metronidazole (1500 mg/day). An ACTH test was performed, which showed 840 nmol/Lat baseline and 895 nmol/L 1 hour after injection, and she was started on treatment with 300 mg/day hydrocortisone.

Four days later, a second set of thoracic, cerebral and abdominal CT scans was performed. In addition to the abscess of thelingula and the drained pyopneumothorax, these scans revealed a large number of small abscesses of the upper and median lobes of the right lung (Fig 1C), a stable appearance of the hepato-splenomegaly and a partial thrombosis of the left internal jugular vein, without any appearance of stroke.

The anaerobic Gram negative bacillus was identified as a *Fusobacterium necrophorum *sensitive to penicillin, and she was treated with penicillin G for 15 days. The patient improved, becoming apyretic and with normal blood pressure. Withdrawal of ventilation was started and the patient was weaned from the the respiratory asssitance after 3 days. She was discharged from the intensive care unit 12 days after admission.

Her DNA was assayed for SNPs previously incriminated in the detection of the pathogen, the inflammatory response and the coagulation cascade (Table 1). We observed functional variations in her TLR 5 gene and two coagulation variations (heterozygous TF603 and PAI-1 4G-4G homozygosity) associated with thrombotic events.

**Table 1:** Genotype findings at all loci tested. Functional variations were observed in the TLR 5 gene, the flagellin receptor, TLR5-F616L, a heterozygous mutation TLR5-R392 and two coagulation variations resulting in thrombotic events (HT TF603 and PAI-1 4G-4G homozygous). Genes encoding proteins involved in inflammation genes were normal.

	Ref	WT/WT	WT/M	M/M			WT/WT	WT/M	M/M
TLR2 R753Q	rs5743708	X			IL 10-1082	rs1800872	X		
TLR5-R392	rs5744168		X		TNF b1/b2	rs2229094	X		
TLR5-N592S	rs2072494	X			TNF 238	rs361525	X		
TLR5-F616L	rs5744174			X	TNF 308	rs1800629	X		
TLR4 D299G	rs4986790	X			TNF 376	rs1800750	X		
Fc-GrIIα	rs1801274	X			PAI-1	rs1799768			X
CD 14	rs2569190		X		Fibrinogen	rs6050	X		
SPD 11	rs721917	X			TF p603	[14]		X	
SPD 160	rs2243639		X		EPCR	rs867186	X		
MIF	rs755622		X		Factor II	rs1799963	X		
IL 6	rs1800795		X		Factor V	Rs6025	X		
IL 10-592	rs1800872		X		Factor VII	Rs6046	X		

Abreviations : Toll like receptor (TLR); Fc receptor for IgG (Fc-gamma RII); macrophage migration inhibitory factor (MIF); Endothelial protein C receptor (EPCR); Tissue factor (TF); Tumor Necrosis Factor (TNF); Surfactant Protein D(SPD); WT: Wild type concerns the frequent allele; M: mutation concerns the rare allele. Ref: reference number for the studied SNP.

## Discussion

Lemierre's syndrome is frequently due to infection with a strictly anaerobic Gram negative bacillus, *Fusobacterium necrophorum*, a saprophyte of the oropharynx, digestive tract and genital pathways. Lemierre's syndrome, which mainly affects young patients without any previous medical history, consists of a combination of fever, shivering, deterioration of the general condition, and pain and/or cervical tumefaction along the sterno-cleido-mastoid muscle, resulting from a suppurative thrombophlebitis of the tonsillar and peri-tonsillar veins that can extend to the internal jugular vein. This rare syndrome usually occurs following a banal pharyngitis. The appearance of pulmonary symptomatology (cough, dyspnea, thoracic pain) and/or abdominal symptomatology (hepato and/or spleno-megaly, hepatalgia, cholestasis and/or biological cytolysis) suggesting septic metastases, points to Lemierre's syndrome and justifies the implementation of a suitable antibiotic regimen. Other septic phenomena, such as arthritis, mediastinitis, meningitis and/or endocarditis, are found more rarely.

The physiopathology of Lemierre's syndrome remains controversial. *Fusobacterium necrophorum *[3] is a commensal of the normal flora of the human oropharynx, digestive tract, genital and urinary pathways and normally does not invade those mucosae. It is not known why *Fusobacterium necrophorum *becomes pathogenic in certain individuals, although a lowering of local pharyngeal defenses following a viral or bacterial infection may encourage this invasion by creating an anaerobic micro environment [4]. Another explanation is based on the capacity of these bacteria to secrete hemolysins, hemagglutinins and leukocidins, resulting in the formation of micro-abscesses by fibrin and platelet aggregation.

The recent discovery of anti-infection defense mechanisms, in particular Toll like receptors, and the finding that these receptors have functional variations open up new avenues in the pathophysiology of the Lemierre's syndrome, as illustrated in this clinical case.

The innate immune response to *Fusobacterium necrophorum *is complex and involves both tissue immunity (cathelicidins and defensins) and cellular immunity (TLR receptors). Although *Fusobacterium necrophorum *is not a flagellate bacterium, it is capable of synthesizing Pilin [5]. Pilin and type IV pili (monomer), may be one of the TLR5 triggers. The TLR5-F616L and TLR5-R392 mutations, which are associated with infections by flagellate bacteria, such as *Legionella pneumophila *[6], may also be associated with *Fusobacterium necrophorum *infections. To date, there is no scientific proof with this assertion.

A second facet of the symptomatology of Lemierre's syndrome is the presence of hypercoagulability, resulting in thrombophilia. A procoagulating mutation of prothrombin was recently associated with this syndrome [7]. Interestingly, we observed a combination of one prothrombogenic and one anti-fibrinolytic variations in our patient. The last one was the 4G-4G homozygous gentotype of the PAI-1 gene which encodes for a primary anti-fibrinolytic molecule. This gentotype is responsible for an increase in plasma concentrations of PAI-1. Moreover, this mutation alone [8] or in combination with other prothrombotic genetic anomalies [9,10] is a risk factor for myocardial infarction and venous thrombo-embolic phenomena. The pathophysiology of sepsis is due to imbalances in the coagulation and fibrinolysis systems, and any factor that accentuates these imbalances can influence the host response. Patients suffering from meningococcemia and whose close relatives were carriers of the 4G-4G genotype were found to be 6 times more likely to develop septic shock rather than meningitis [11]. Moreover, this genotype was also found to be associated for an increase in the mortality of patients suffering from multiple injuries [12] and meningococcemia [13]. This SNP can therefore partly explain the physiopathology of Lemierre's syndrome, as well as the severity of the clinical picture in our patient. Similarly, the variation in tissue factor promoter results in a spontaneous and induced overexpression of this trigger of coagulation during sepsis. Of course we have assayed a selection of previously reported SNPs, but many other potentially important reported candidate SNPs have not been examined.

## Conclusion

To our knowledge, this article is the first to identify SNPs in a patient suffering from Lemierre's syndrome. The innate immune response and the prothrombogenic mutations observed in this patient could explain, at least in part, the symptoms of Lemierre's syndrome. Genomic study of several patients with Lemierre's syndrome may help to reveal its complex pathophysiology.

## Abbreviations

SNP: Single nucleotide polymorphism

TLR 5: Toll like 5 receptor gene

TF: Tissue Factor

PAI-1: Plasminogen-Activator-Inhibitor-1

PEEP: Positive end expiratory pressure

Fc-GRII: Fc receptor for IgG

MIF: macrophage migration inhibitory factor

EPCR: Endothelial protein C receptor

TNF: Tumor Necrosis Factor

SPD: Surfdactant Protein D

## Competing interests

The author(s) declare that they have no competing interests.

## Authors' contributions

JMC, JPM and RG drafted the manuscript, and FG, OL, JPR, HL oversaw the sections on infectious disease. All authors read and approved the final manuscript.

## Pre-publication history

The pre-publication history for this paper can be accessed here:


